# Correlating pharmaceutical data with a national health survey as a proxy for estimating rural population health

**DOI:** 10.1186/1478-7954-8-25

**Published:** 2010-09-14

**Authors:** Ronald E Cossman, Jeralynn S Cossman, Wesley L James, Troy Blanchard, Richard Thomas, Louis G Pol, Arthur G Cosby

**Affiliations:** 1 Social Science Research Center, Mississippi State University, Mississippi State, Mississippi, USA; 2 Social Science Research Center and the Department of Sociology and Social Work, Mississippi State, Mississippi, USA; 3 Department of Sociology, University of Memphis, Memphis, Tennessee, USA; 4 The Department of Sociology, Louisiana State University Baton Rouge, Louisiana, USA; 5 University of Tennessee Health Science Center, Memphis University of Tennessee Health Science Center, Memphis, Tennessee, USA; 6 College of Business Administration, University of Nebraska, Omaha, Nebraska, USA

## Abstract

**Background:**

Chronic disease accounts for nearly three-quarters of US deaths, yet prevalence rates are not consistently reported at the state level and are not available at the sub-state level. This makes it difficult to assess trends in prevalence and impossible to measure sub-state differences. Such county-level differences could inform and direct the delivery of health services to those with the greatest need.

**Methods:**

We used a database of prescription drugs filled in the US as a proxy for nationwide, county-level prevalence of three top causes of death: heart disease, stroke, and diabetes. We tested whether prescription data are statistically valid proxy measures for prevalence, using the correlation between prescriptions filled at the state level and comparable Behavioral Risk Factor Surveillance System (BRFSS) data. We further tested for statistically significant national geographic patterns.

**Results:**

Fourteen correlations were tested for years in which the BRFSS questions were asked (1999-2003), and all were statistically significant. The correlations at the state level ranged from a low of 0.41 (stroke, 1999) to a high of 0.73 (heart disease, 2003). We also mapped self-reported chronic illnesses along with prescription rates associated with those illnesses.

**Conclusions:**

County prescription drug rates were shown to be valid measures of sub-state estimates of diagnosed prevalence and could be used to target health resources to counties in need. This methodology could be particularly helpful to rural areas whose prevalence rates cannot be estimated using national surveys. While there are no spatial statistically significant patterns nationally, there are significant variations within states that suggest unmet health needs.

## Background

Chronic diseases exact a toll on the population, yet most national surveillance systems addressing the level of prevalence lack the geographic detail necessary to allow public health officials to intervene effectively in terms of health services allocation, especially in rural areas. Health officials must depend on data from the National Health Interview Survey (NHIS), the National Health and Nutrition Examination Survey (NHANES), and the Behavioral Risk Factors Surveillance System (BRFSS) to calculate the nationwide prevalence of chronic illnesses [1], although the limitations of these surveys for measuring minority populations are well-known [2,3]. Due to the nature of survey design, statistics cannot be derived for rural areas, although data for selected metropolitan areas have been made available [4]. Given survey data limitations, the population of nearly two-thirds of US counties is excluded from the sample population. Shifting disease surveillance to the county level with county data has the potential to create a surveillance system that more accurately characterizes the public health burden of chronic illnesses, identifies high-concentration areas, improves health care resource targeting, and advances disease prevention and control at a localized level.

The achievement of national health goals is directly tied to the ability to target intervention strategies to people residing in specific geographic areas [5]. The higher the data resolution, the more effectively resources could be allocated. Most adults diagnosed with heart disease, high blood pressure, and diabetes report taking prescription medication for their illnesses (heart disease -- 81% in 1987 and 77% in 2001; high blood pressure -- 94% in 1987 and 97% in 2001 [6]; diabetes -- 83% in 1987 and 93% in 2001 [6], 85% in 2001 [7] or 83% in 2001-2002 [8], depending on the survey). Reliable prescription data at the sub-national level could be a valid proxy measure for the prevalence of these chronic illnesses. We tested the viability of using data on prescriptions filled as a proxy measure for illness prevalence rates by comparing prescriptions-filled rates with state-level BRFSS data, using population estimates to supplement survey estimates.

As a point of clarification, cancer, the second-leading cause of death in the US, was not one of the selected chronic conditions in the prescriptions-filled dataset. Most cancer drugs are used in hospitals, clinics, and physician offices and thus would not reflect the residence of individuals with cancer, a central feature of this research.

The Dartmouth Atlas of Health Care in the United States is the most comprehensive study of smaller area geographic variation in diseases, but it was carried out at the level of Hospital Service Area and Hospital Referral Regions [9-11]. A study similar to the Dartmouth Atlas examined prescription drug use in Michigan but did not address major chronic disease prescription medications in particular [12]. Despite significant regional variations found in a study of Medicare data for a group of male Hispanics experiencing renal failure, data constraints mean that geography typically gets introduced into research models only as a consideration for rural versus urban populations [13]. Even the rural-urban distinction yields important insights for medically underserved populations. Rural African Americans are less likely to control their diabetes and hypertension than their urban counterparts, and American Indians have significant regional variations in risk and prevalence of diabetes [14-16]. Recognizing spatial differences in diabetes treatment, the National Institute of Diabetes and Digestive and Kidney Diseases (NIDDK) is funding research on regional variations in health outcomes among diabetic minorities [17], and the Agency for Healthcare Research and Quality is funding research on geographic patterns in recurrent strokes [18].

Some state-specific studies have been produced, although they differ from the depth of analysis that we propose. For example, the Prescription Drug Atlas [19] describes the geographic distribution of a number of drug classes, based on a convenience sample of Express Script plan customers. The Centers for Disease Control and Prevention (CDC) results from the 1988 and 1989 BRFSS revealed the predominance of states with high diabetes rates grouped east of the Mississippi River, with none of the highest-rate states in the West [20,21]. More recently, CDC researchers decomposed the BRFSS to 100 metropolitan statistical areas (MSA) to examine the prevalence of Type 2 diabetes [22]. Researchers from the State Center for Health Statistics in North Carolina used results from their 10 most populous counties and then aggregated the remaining 90 counties into three regions [23]. A recent review of the Indicators for Chronic Disease Surveillance noted that the current system is restricted to state and national levels of geography for surveillance, confirming this lack of county-level specificity [24].

Our population-level methodology overcomes some problems found in state-level studies. As Geiss et al noted, "Data from population-based studies are generally considered more reliable than data from selected groups within the population because the latter may not represent the community with respect to factors such as age and health status" [25]. The full dimensions of the relationship between diagnosis and prescription drug treatment are unknown, but a significant proportion of the diagnosed population can be identified via prescription drug use.

We mapped chronic illness prevalence at a finer geographic scale than MSA or hospital region using prescription data. Statistical analysis using health data mapping enables social and medical scientists to more accurately identify and display areas with high and low disease prevalence rates. This methodology cuts across nominal data categories to potentially reveal cross-sectional and longitudinal geographic patterns and clusters that are otherwise masked. Spatially based chronic disease prevalence data could also be used to address the critical issue of racial and ethnic disparities in health and health care at the county level [26,27].

## Methods

We used one dataset to select the prescription drugs of interest, a different dataset of prescriptions filled at the county level as a proxy measure for chronic diseases, a third dataset to perform an age truncation with a national medical care survey, and a final dataset to calculate correlations as a means of validating the prescription data measure.

IMS Health, Inc., collected prescription drug data from nearly 30,000 suppliers covering 225,000 sites (e.g., drug manufacturers, wholesalers, retailers, pharmacies, mail order, long-term care facilities, and hospitals). A fuller description of IMS Health's products was previously published [28]. To determine the prescription drug classes appropriate for major chronic diseases, we used IMS Health's National Disease and Therapeutic Index (NDTI), a database derived from an ongoing office-based physician panel providing national-level estimates of disease and treatment patterns for office-based physicians. (We chose not to use an expert pharmacy panel to avoid training or practice bias.) The data in NDTI captured all medications associated with a patient visit for a particular treatment. The leading therapeutic classes (also known as Uniform System of Classification or USC) used for the three diseases were identified from this dataset.

The USC classes chosen for diabetes were: (USC 39211) Sulfonylureas, (USC 39220) Biguanides, and (USC 39230) Insulin sensitizers. (Specifically, the drug types included: animal insulins, human insulins, human insulin analogues, sulfonylureas, meglitinides, amino acid derivatives, biguanides, insulin sensitizers, alpha-glucosidase inhibitors, diabetes therapy combinations, and diabetic accessories.)

The classes chosen for heart disease were: (USC 31100) Renin Angiotensin Systemic Antagonist, (USC 31400) Beta and Alpha blockers, and (USC 32000) Cholesterol reducers and Lipotropics. (Specifically, these included Angiotensin-Converting Enzyme (ACE) inhibitors (along with diuretics and other), angiotensin II type I receptor antagonist (alone and in combination), peripheral vasodilators, calcium blockers, beta blockers, alpha-blockers, beta/alpha blockers (with diuretics), alpha blockers (alone and in combination), central acting agent (alone and in combination), antihypertensive (other), HMG-COA reductase inhibitor (3-hydroxy-3methylgluatryl coenzyme A reductase), bile acid sequestrants, fibric acid derivative, cholesterol absorption inhibitor, cholesterol red combination, lipotropics, and antihyperlipidemic agent (other).

The classes chosen for cerebrovascular disease were: (USC 11110) Anticoagulants, (USC 11200) Antiplatelets, and (USC 20200) Seizure disorders. (Specifically, the drug types included: anticoagulant (oral), unfractionated heparins, fractionated heparins, heparines for flushing, injected anticoagulants (other), antiplatelets (oral and injected), fibrinolytic, Vitamin K & related (oral and injected), hemo mod other (injected, oral, topical), l-dopa, antiparkinson (other), movement disorders (other), seizure disorders, anti-ALS, Alzheimer-type dementia, and neurological disorders (other).

Based on the results from the NDTI, we purchased monthly, county-level prescriptions-filled data for 1999-2003 for the chosen drug classes in IMS Health's Xponent database. Total US prescription sales were determined from IMS Health's independently sourced Drug Distribution Data and obtained from pharmaceutical manufacturers, drug wholesalers, and chain warehouses, reported at the individual outlet level. This count included 72% of all prescriptions filled at the individual retail level (February 2006), a stable rate since 1998 to the present. To estimate the remaining 28%, IMS weights retail data to generate estimates representing total dispensed prescription volume at the national, sub-national, and prescriber level. IMS used volume data and pharmacy distance measures to determine applicable weights for sample pharmacies to estimate the dispensed prescription volume for each nonsample pharmacy. Weights were derived through an IMS Health proprietary, patented geo-spatial methodology (personal communication, Stuchlak W: Senior Principal, IMS Management Consulting, IMS Health, Plymouth Meeting: March 31, 2006; PA).

According to IMS Health, retail pharmacies account for 67% of total national prescription sales for therapeutic categories used in the treatment of chronic illnesses. About 23% of sales occur via mail, 8% occur in clinics, long-term care, prisons, universities, and nonfederal hospitals, and 2% occur within federal facilities (e.g., Veterans Administration) [29].

The prescription dataset does not contain patient demographics. To place the prescription data on an equal footing with the BRFSS, which surveys only adults, we determined the percentage of those under 18 who receive diabetes medications (the medication most likely among the three to be taken by children). We used the National Ambulatory Medical Care Survey (NAMCS), a national probability sample survey of visits to office-based physicians conducted by the National Center for Health Statistics [30]. In addition to patient demographics, NAMCS collects data on the therapeutic class of drug prescribed. We aggregated 2000-2002 files to Census-regions level. The denominator, age 18+ state resident population, was derived from the US Census Bureau's inter-censal population estimates. For a full explanation of the methodology, see "Methodology for the State and County Total Resident Population Estimates (Vintage 2009): April 1, 2000 to July 1, 2009" [31]. Among patients under age 20 (the closest age cut-point), the usage of diabetes medications was always below 1%, indicating that children could be dropped from the base population.

A rolling 12-month average was calculated to smooth the rates and to account for multiple-month prescription fills. The refined rate of prescriptions-filled calculation is:

$$\frac{\begin{array}{c} (Estimated\;prescriptions\;by\;category\\ filled\;in\;a\;calendar\;year\;in\;the\;state)\;/\;12\;months \end{array}}{Age\;18\;and\;over\;resident\;state\;population\;\left(in\;100s\right)}$$

We did not attempt to adjust for the three-month order because it was not widely available in 1999-2003. We cannot fully account for those who only fill their prescriptions for only part of the year beyond our rolling 12-month calculation. As an example, in 2003, prescriptions in the retail channel, which were overwhelmingly 30-day supplies at the time, accounted for 86% of all prescriptions for the Angiotensin Receptor Blocker drug class. Mail order prescriptions, which are typically for 90 days, only accounted for 10% of ARB prescriptions, and long-term care accounted for the remaining 4% of ARB prescriptions.

BRFSS, a state-level monthly telephone survey of adults about behaviors associated with health risks and the incidence of medical conditions, provided our reference point [32]. BRFSS data have been routinely used to create state-level estimates of chronic illness, health risks, and national prevalence rates, and more recently, for metropolitan and micropolitan area prevalence rates; however, due to the survey design, the BRFSS continues to undersample rural residents. We used the following three survey questions from the 1999-2003 BRFSS: "Have you ever been told by a doctor, nurse or other health professional that you had: (i) diabetes, (ii) coronary heart disease or (iii) high blood pressure?"

An important shortcoming of existing survey data is that they are not spatial in nature. According to Stan Openshaw, an early leader in exploratory data visualization: "People DIE each year because no one BOTHERS to properly analyze DISEASE and DEATH data for unusual localized concentrations" [[33], emphasis in original]. Maps are often used to display health information, but typically there is an inadequate effort to empirically identify spatial patterns or apply spatial statistics to test hypotheses. We mapped and compared state-level results from BRFSS and IMS data; IMS data were also mapped at the county level. After visually assessing maps presented here, we conducted a statistical test of spatial autocorrelation to test whether prescriptions-filled rates were equally likely to occur at any location, using the Global Moran's I [34].

## Results

### Introduction

Direct comparisons between the state-level IMS Health prescription rates and the state-level BRFSS disease prevalence rates are restricted by the number of states reporting to BRFSS in each year. Only the diabetes question was asked in all states in all five years. We paired drug classes with appropriate BRFSS questions (Table 1). The statistically significant correlations ranged from a low of 0.41 to a high of 0.73 for years in which all states reported.

**Table 1:** Correlations of State-level BRFSS to State-level Rx

Disease Question vs. Prescription Matched	1999	2000	2001	2002	2003
BRFSS Q: "Has a doctor, nurse, or other health professional ever told you that you have angina or coronary heart disease?" vs. Heart disease prescriptions filled	*0.438*^*a*^	--	*0.662*^*a*^	--	*0.613*^*a*^
N (states)	*21*	14	*20*	7	*24*
BRFSS Q: "Have you ever been told by a doctor, nurse or other health professional that you have high blood pressure?" vs. Heart disease prescriptions filled	*0.537*^*a*^	--	*0.635*^*a*^	--	*0.733*^*a*^
N (50 states plus District of Columbia)	51	6	51	11	51
BRFSS Q: "Have you ever been told by a doctor, nurse or other health professional that you have high blood pressure?" vs. Stroke prescriptions filled	*0.406*^*a*^	--	*0.499*^*a*^	--	*0.622*^*a*^
N (50 states plus District of Columbia)	51	6	51	11	51
BRFSS Q: "Have you ever been told by a doctor that you have diabetes?" vs. Diabetes prescriptions filled	*0.588*^*a*^	*0.609*^*a*^	*0.625*^*a*^	*0.695*^*a*^	*0.713*^*a*^
N (50 states plus District of Columbia)	51	51	51	51	51

^*a *^= *Correlation is significant at the 0.01 level (2-tailed test)*

-- Data unavailable;

Note: Correlations with populations of less than 30 are unstable and should be viewed with caution

The correlation between self-reported diabetes and diabetes prescriptions was consistently high (Table 1). The correlation between self-reported high blood pressure and heart disease prescriptions was slightly higher than the correlation between self-reported high blood pressure and stroke prescriptions. While the correlation was high and statistically significant, only a small number of states have reported. Therefore, these results should be viewed with caution.

### Diabetes

When comparing the state-level IMS Health prescription rates with the state-level BRFSS diabetes prevalence rates, the correlations ranged from 0.59 to 0.71 (p < .01, Table 1.) We excluded gestational diabetes (<1% of national cases) as a temporary condition [35]. The BRFSS mean state-level adult prevalence rate ranged from 5.6% to 7.1%, while the state-level adult prescriptions-filled rates for diabetes medications were roughly half that. When the prescription rates were further disaggregated to the county level, the average county rate of diabetes prescriptions filled ranged from 2.2% to 2.7%, although 20% of US counties were in the top quintile of 4% to 18%. The ratio of the prescriptions-filled rates to the BRFSS prevalence rates was relatively stable across time, at 0.6.

Although the two diabetes prevalence rates differ (7% vs. 4%), they are fundamentally different measures and were not expected to be equal. In the case of diabetes, according to the NIDDK, the combined US prevalence rate for Type 1 and Type 2 diabetes is 7% (in 2003); of that, 95% was Type 2, which yields a Type 2-only prevalence rate to 6.8%. Among these cases, 70% were diagnosed, yielding an estimated Type 2 prevalence rate of 4.7%. Among the remaining cases, only 85% were drug-treated, yielding an estimated Type 2 prevalence rate of 4% [36]. This estimated prevalence percentage was very close to the level produced by our prescription drug methodology (4.1% vs. 4.0%)(Table 2). Additionally, a study similar to ours in Portugal found this prescription methodology slightly underestimated diabetes prevalence compared to a national health survey [37]. Double counting of individuals via the total number of prescriptions filled (e.g., counting two different prescription drugs as two different individuals) would be unlikely because almost 90% of individuals in drug therapy are receiving monotherapies or a single drug treatment [38].

**Table 2:** Correlations of State-level BRFSS to State-level Prescription Drug Filled

	1999	2000	2001	2002	2003
BRFSS Q: "Have you ever been told by a doctor that you have diabetes?" correlated with diabetes prescriptions filled	*0.588*^*a*^	*0.609*^*a*^	*0.625*^*a*^	*0.695*^*a*^	*0.713*^*a*^
N (50 states plus the District of Columbia)	51	51	51	51	51
BRFSS Diabetes (State-level):					
Mean^b^	5.59%	6.05%	6.45%	6.66%	7.14%
Standard Deviation	0.95%	0.87%	1.24%	1.20%	1.31%
Prescriptions-Filled (State-level):					
Mean	3.38%	3.55%	3.76%	3.99%	4.13%
Standard Deviations	0.70%	0.73%	0.74%	0.78%	0.79%
Ratio of Rx/BRFSS rates	0.62	0.60	0.60	0.62	0.59
Prescriptions-Filled (County-level):					
Mean	2.22%	2.33%	2.47%	2.62%	2.73%
Standard Deviation	1.34%	1.41%	1.49%	1.59%	1.64%
N (counties)	3,103	3,103	3,103	3,103	3,103

^*a *^= *Correlation is significant at the 0.01 level (2-tailed)*

^b ^= Mean of the state-level rates, not a computed national rate.

**Figure 1:**
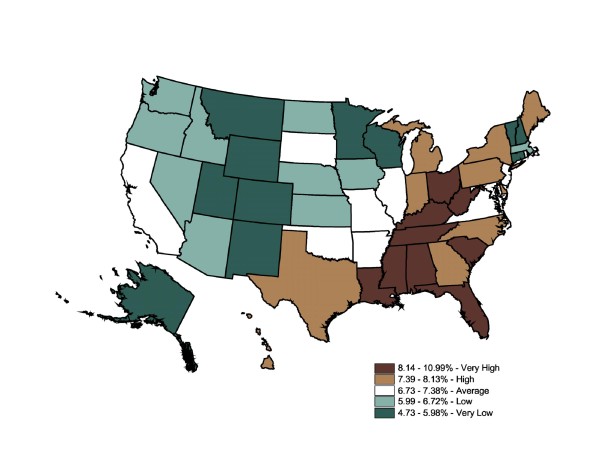
**State-Level Percentage of the Adult Population Told by a Doctor They Have Diabetes (BRFSS), 2003**. Note 1: Behavioral Risk Factor Surveillance System is used in the map. Note 2: Interpreted as the percent of the adult population told by a doctor they have diabetes in 2003. Note 3: 50 states and DC reporting. Note 4: Population 18+ is shown.

We used quintiles to facilitate comparisons between relative differences in diabetes prevalence between the BRFSS and prescriptions-filled maps. The color ramp was based on the ColorBrewer color diagnostic tool [39]. Figure 1 displays mapped BRFSS data, with 10 states in each category except for the dark green category with 11. The self-reported prevalence rates range from 4.7% to 11%. This map shows 10 states ranked "very high" in diabetes prevalence rates (WV, OH, KY, TN, SC, FL, AL, MS, LA, and DC). The remainder of the South ranks as an area of "high" prevalence (7.4% - 8.1%). Strikingly, the Midwest and West were generally "very low" (4.7% - 6%) and "low" (6% - 6.7%). Overall, BRFSS mapped state-level data suggest diabetes prevalence rates were divided into high-prevalence East and low-prevalence West zones, with a cluster of "very high" states roughly following the Appalachian mountain region. The Global Moran's I for Figure 1 is 0.57, which indicates a relatively low degree of positive spatial autocorrelation (ie, the spatial pattern is not random) (p = 0.001). A positive spatial autocorrelation (+0.5 to +1) indicated neighboring counties were more like each other than distant counties. Conversely, a negative spatial autocorrelation (-0.5 to -1) indicated the rates in neighboring counties were unlike each other. Scores of -0.4 to +0.4 suggest weak autocorrelation, with a score of 0.0 indicating no spatial autocorrelation at all, a totally random geographic distribution.

**Figure 2:**
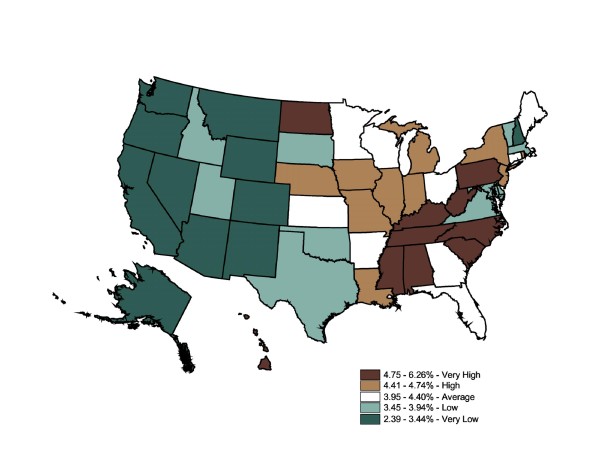
**State-Level Percentage of the Adult Population Who Filled Diabetes Prescriptions, 2003**. Note 1: IMS Health Xponent is used in the map. Note 2: Interpreted as the percent of the adult population who filled prescriptions in 2003. Note 3: 50 states and DC reporting. Note 4: Population 18+ is shown.

The IMS Health state-level prescription data map (Figure 2) was generally similar to the BRFSS map. Though the range for categories was not equal (BRFSS ranges 4.7% - 11% vs. Rx ranges 2.4% - 6.3%), quintiles allowed for a map-to-map comparisons of the relative top 20%, the next highest 20%, etc. The prescriptions-filled map displayed the same cluster of high rates in the East, specifically the South, while lower rates were clustered in the Midwest and West. Since these were relative rankings based on a national average, a state might move from one quintile in one map to an adjacent quintile in another map, although this move might not be statistically significant. Six states shifted down two quintiles across the two datasets (OH, FL, DC, TX, DE, and CA), and six states shifted up by two or more quintiles (ND, NE, IA, MN, WI, and CT). For the first group, the differences between disease prevalence and drug treatment could not be attributable to underdiagnosis because the individuals reported that they had been told by a medical professional that they had the chronic illness. For the second group, while self-reported prevalence was relatively low in these states, a fairly large number of prescriptions were filled in these states. Medications could be overprescribed to residents, or nonresidents were filling prescriptions in that region at a higher rate than in other states; alternatively, these states may have perfectly acceptable prescription-fill rates, but other states could be underprescribing. Further investigation would be necessary to support either conclusion. The Global Moran's I for Figure 2 is 0.50, which indicates a relatively low degree of positive spatial autocorrelation (ie, the spatial pattern is not random) (p = 0.001). The prescriptions-filled map contains the same degree of spatial autocorrelation as the BRFSS state map (0.50 vs. 0.57).

**Figure 3:**
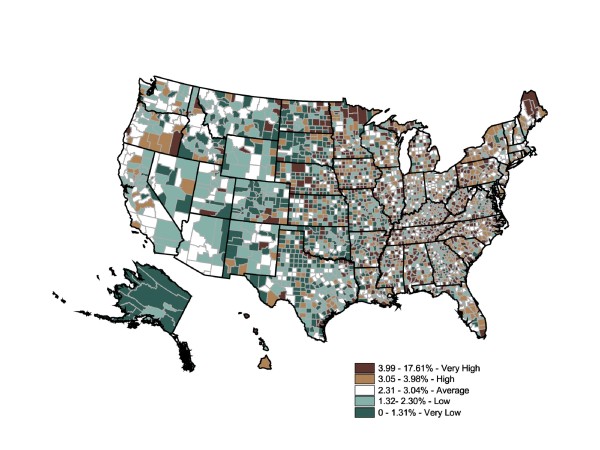
**County-Level Percentage of the Adult Population Who Filled Diabetes Prescriptions, 2003**. Note 1: IMS Health Xponent is used in the map. Note 2: Interpreted as the percent of the adult population who filled diabetes prescriptions in 2003. Note 3: 50 states and DC reporting. Note 4: Population 18+ is shown.

The advantage county-level prescription data offer is the ability to inspect intrastate and regional differences in prescription rates. Figure 3 maps refined rates of filled diabetes prescriptions at the county level for the year 2003. We used the same colors and standards of quintile categories as the BRFSS map to facilitate comparisons among the BRFSS state map, the prescriptions-filled state map, and the prescriptions-filled county map. The Figure 3 map shows a much more varied distribution of diabetes prevalence (expressed by fill rates) and a possible disconnect between prescription fill rates and geographic estimates of diabetes prevalence from mapped BRFSS data in Figure 1. When individual county rates were calculated and mapped, the range of prescriptions-filled rates expanded to 0% to 17.61% (two counties were considered outliers: Adams County, ND, has a diabetes prescription-fill rate of 17.61%, while Montour County, PA, has a rate of 13.97%. The remainder of the counties in this top quintile falls between 3.99% and 10.76%.).

Striking prescription-fill rate differences were found within the "very high" prevalence states. One reason for the markedly varied sub-state levels is that county-level population calculations do not permit urban counties to overwhelm the rural counties. Another explanation for the variety was that prescription-fill rates vary dramatically within states and regions. Therefore, using state-level data alone may be insufficient to appropriately target areas with high or low prevalence.

Three interesting patterns are noteworthy. First, counties with relatively low prescriptions-filled rates in states with overall high rates of prevalence may identify clusters of undermedication. Likewise, counties with relatively high prescriptions-filled rates within states with relatively low prevalence rates were similarly identified as sites for further investigation. Finally, adjacent counties where the rates differ by two or more categories (e.g., dark brown adjacent to light green) may suggest fundamental differences between the two counties in either the resident population or health care delivery system--or reflect concentrations of residents crossing county lines for their prescriptions. In regard to this last pattern, Iowa was an excellent example. While at the state-level Iowa had a relatively low self-reported prevalence and "average" rate of prescriptions filled, it showed a great variety among counties, sometimes even in adjacent counties. While the county-level map generated using prescriptions-filled data matches the general pattern of the state-level BRFSS map, it also displayed potentially important regional differences as well as significant levels of intercounty variation. Planning appropriate health care interventions for diabetics would more likely be improved by using the county-level rather than the state-level map. Shifting the scale from the state level to the county level changes the spatial statistic dramatically. The Global Moran's I for Figure 1 is 0.0947, which indicates an almost total absence of spatial autocorrelation (ie, the spatial pattern is random) (p = 0.001).

### Heart Disease

We compared coronary heart disease (BRFSS) to the broader category of heart disease prescriptions filled (coronary heart disease is a subset of all heart diseases; to avoid confusion in this article, *coronary *is set off in italics). Diseases of the heart are the leading cause of death in the US, responsible for 29% of deaths in 2001 and 2002 [40]. In 2003, cardiovascular disease was an underlying cause of death in 37% of all cases and an underlying or contributing cause of death in 58% of all deaths [41]. From 1992-2002, 1 in 3 adults was estimated to have cardiovascular disease, roughly 71 million people [42]. Another study (1999-2000) estimated prevalence of cardiovascular diseases to be 34% of the total population [43]. The estimated prevalence of *coronary *heart disease (in 1999-2000) was 7% of the total population and 8% for males [44]. Thus, the broad category of heart disease may be roughly 33% of the population, while *coronary *heart disease is estimated in 7% of the population. But the definitions used to measure these diseases have changed several times (e.g., International Classification of Diseases, revisions 6-10), so precise trend studies are difficult to produce. Additionally, national prevalence rates are derived from sample-based surveys such as the NHANES and the BRFSS [45]. Surveys are subject to sampling variability as well as survey design flaws, respondent classification errors, data processing mistakes, and poor coverage of the population. Specifically, we were concerned about the inability of survey data to estimate small area disease prevalence, particularly in rural areas where individuals were not surveyed.

If cardiovascular disease was eliminated as a cause of death, life expectancy at birth would increase nearly seven years [46]. Of all deaths attributed to heart disease, 17% occurred in people under age 65 and were considered premature deaths [47]. "Reducing premature death from heart disease and eliminating disparities will require preventing, detecting, treating, and controlling risk factors for heart disease in young and middle-aged adults" [48]. Ironically, the leading cause of death was not tracked in every state, in every year via the BRFSS, which was conducted by each state. Just 24 states administered this health question in 2003.

The state-level BRFSS *coronary *heart disease prevalence rates correlate with the state-level IMS Health heart disease prescription rates between 0.44 to 0.61 (p < .01, Table 3.). These correlations were based on data from 20 to 24 states, and correlations based on samples of fewer than 30 may be unstable. The BRFSS mean state-level adult prevalence rate for *coronary *heart disease is roughly 4.4%; the state-level adult heart disease prescriptions-filled rate ranged from 15.2% (1999) to 18.7% (2003). When the prescriptions were reported at the county level, the average rate was 9.4% (1999) to 11.9% (2003). Nearly 1 in 6 adults fills orders for heart disease prescription drugs, half of estimated prevalence rates. The ratio of the prescriptions-filled rates to BRFSS prevalence rates range from a low of 1.6 to 2.3.

**Table 3:** Correlations of State-level BRFSS to State-level Rx

	1999	2000	2001	2002	2003
BRFSS Q: "Have you ever been told by a doctor that you have coronary heart disease?" compared to heart disease prescriptions filled	*0.438*^*a*^	--	*0.662*^*a*^	--	*0.613*^*a*^
N (states)	21	14	20	7	24
BRFSS Heart Disease (State-level):					
Mean^b^	4.27%	--	4.47%	--	4.48%
Standard Deviation	0.90%	--	1.08%	--	1.39%
Prescriptions-Filled (State-level):					
Mean	15.19%	16.04%	16.99%	18.02%	18.74%
Standard Deviations	3.44%	3.57%	3.64%	3.83%	3.96%
Ratio of Rx/BRFSS rates	1.60	--	1.57	--	2.25
Prescriptions-Filled (County-level):					
Mean	9.41%	10.01%	10.60%	11.28%	11.85%
Standard Deviation	5.92%	6.33%	6.64%	7.05%	7.32%
N (counties)	3,103	3,103	3,103	3,103	3,103

Note: Correlations with populations of less than 30 are unstable and should be viewed with caution.

^*a *^= *Correlation is significant at the 0.01 level (2-tailed)*

^b ^= Caution: Mean of the state-level rates, not a calculated national rate

-- Data unavailable

**Figure 4:**
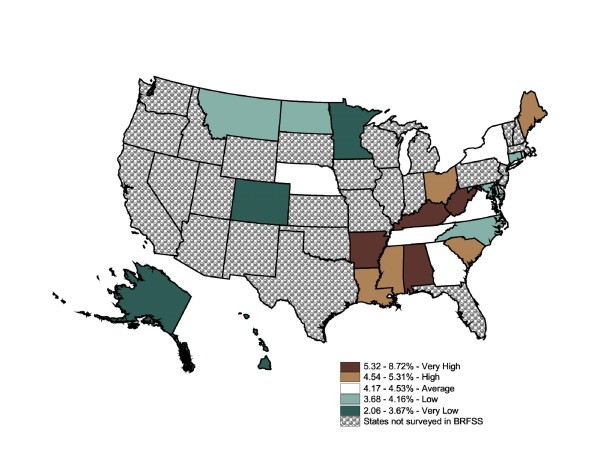
**State-Level Percentage of the Adult Population Told by a Doctor They Have Coronary Heart Disease (BRFSS), 2003**. Note 1: Behavioral Risk Factor Surveillance System is used in the map. Note 2: Interpreted as the percent of the adult population told by a doctor they have coronary heart disease in 2003. Note 3: 22 states and DC reporting. Note 4: Population 18+ is shown.

The BRFSS self-reported prevalence ranged from 2.06% to 8.72% (Figure 4). The map contains five states in each category except for the top 20% category. This map shows four central southern states ranked "very high" in *coronary *heart disease prevalence rates (5.3%+); the remainder of the South ranks as an area of "high" or "average" prevalence (4.2% - 5.3%). The majority of states in the Midwest and West did not report this question, although half of the eight "low" and "very low" prevalence states were in the Midwest and West. The BRFSS mapped state-level data suggests *coronary *heart disease prevalence rates were divided into high-prevalence Southeast and low-prevalence Midwest and West zones, with a cluster of very high-prevalence states that roughly follow the Appalachian mountain region.

**Figure 5:**
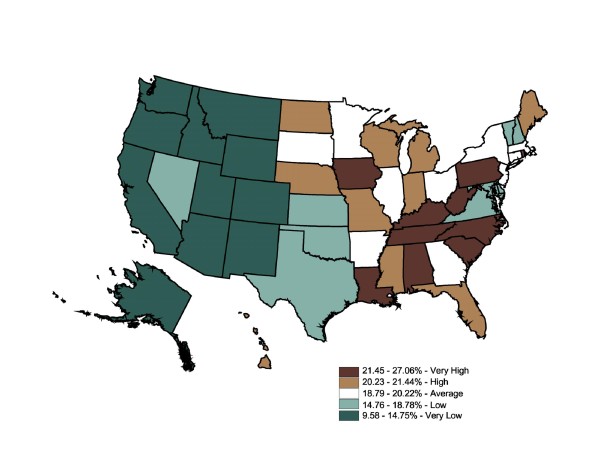
**State-Level Percentage of the Adult Population Who Filled Heart Disease Prescriptions, 2003**. Note 1: IMS Health Xponent is used in the map. Note 2: Interpreted as the percent of the adult population who filled heart disease prescriptions in 2003. Note 3: 50 states and DC reporting. Note 4: Population 18+ is shown.

In comparison, the IMS Health state-level prescription data map (Figure 5) filled in the missing states, completing the health picture of the Midwest and West. It also reinforced the same cluster of high rates in the Southeast, while low rates were clustered in the Midwest and West. Arkansas reported in the "very high" quintile of *coronary *heart disease, but was ranked "average" for heart disease prescriptions filled, perhaps suggesting residents were undertreated via drugs for heart disease. Five states shifted by two or more categories. Since these categories are based on relative (state-to-state) measures, it was not possible to link actual need for prescriptions to prescription rates in each state. Rather, it suggested which states warranted further investigation. The Global Moran's I for Figure 5 is 0.56 (p = 0.001), which indicates a low level of positive spatial autocorrelation.

**Figure 6:**
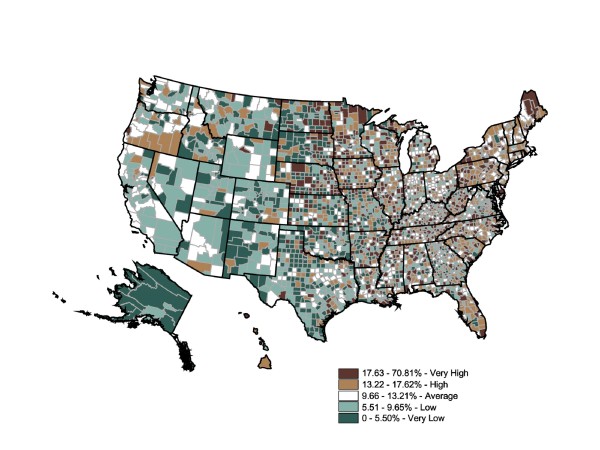
**County-Level Percentage of the Adult Population Who Filled Heart Disease Prescriptions, 2003**. Note 1: IMS Health Xponent is used in the map. Note 2: Interpreted as the percent of the adult population who filled heart disease prescriptions in 2003. Note 3: 50 states and DC reporting. Note 4: Population 18+ is shown.

The Figure 6 map shows a much more varied distribution of heart disease prevalence (expressed by fill rates) along with a possible disconnect between prescription fill rates and the geographic estimates of *coronary *heart disease prevalence from mapped BRFSS data in Figure 4. The range of rates was expanded to 0% to 70.8% (although two counties in which the rate was 50% or more were interpreted as regional distribution centers. The reported heart disease prescription-fill rate is 70.81% for Adams County, ND, and 56.16% for Montour County, PA. Without further investigation, we assume these to be prescription distribution centers. The remainder of the counties falls under 50%.).

Striking intercounty differences in prescription fill rates were found within the "very high" prevalence states as well as the "very low" prevalence states. Again, the most informative patterns would be low prescription-fill-rate counties in states with overall high prevalence rates, high prescription-fill-rate counties in states with overall low prevalence rates, and "very high" or "high" prescription-fill-rate counties adjacent to "low" or "very low" prescription-fill-rate counties. Shifting scale from the state to county level changes the spatial statistic dramatically. The Global Moran's I for Figure 6 is 0.08 (p = 0.001), which indicates a near absence of spatial autocorrelation.

### Stroke

Similar to *coronary *heart disease versus general heart disease, stroke medications are used to treat a fraction of hypertension cases. Thus, we would expect that the prescription rates would be lower than the broader self-reported rates of hypertension. Correlations for state-level BRFSS self-reported hypertension prevalence rates to state-level IMS health stroke prescription rates ranged from 0.41 to 0.62 (p < .01, Table 4.) The BRFSS mean state-level adult hypertension prevalence rate ranged from 24.3% to 25.6%, while the state-level adult stroke prescriptions-filled rate ranged from 3.4% to 4.6%. When the prescription rates for counties were calculated, the average rate dropped from 1.9% to 2.7%. The ratio of the prescriptions-filled rates to the BRFSS prevalence rates ranged from 0.15 to 0.19, remaining relatively stable across time.

**Table 4:** Correlations of State-level BRFSS to State-level Rx

	1999	2000	2001	2002	2003
BRFSS Q: "Have you ever been told by a doctor that you have hypertension?" compared to Stroke prescriptions filled	*0.406*^*a*^	--	*0.499*^*a*^	--	*0.622*^*a*^
N (50 states plus the District of Columbia)	51	--	51	--	51
BRFSS Hypertension (State-Level):		--		--	
Mean^b^	24.32%	--	25.69%	--	25.56%
Standard Deviation	3.22%	--	2.76%	--	3.34%
Prescriptions-Filled (State-level):					
Mean	3.44%	3.61%	3.91%	4.29%	4.64%
Standard Deviations	0.69%	0.73%	0.77%	0.87%	0.96%
Ratio of Rx/BRFSS rates	0.15	--	0.16	--	0.19
Prescriptions-Filled (County-level):					
Mean	1.91%	2.00%	2.18%	2.41%	2.67%
Standard Deviation	1.45%	1.53%	1.66%	1.82%	1.99%
N (counties)	3,103	3,103	3,103	3,103	3,103

^*a *^= *Correlation is significant at the 0.01 level (2-tailed)*

^b ^= Mean of the state-level rates, not a calculated national rate

-- Data unavailable

**Figure 7:**
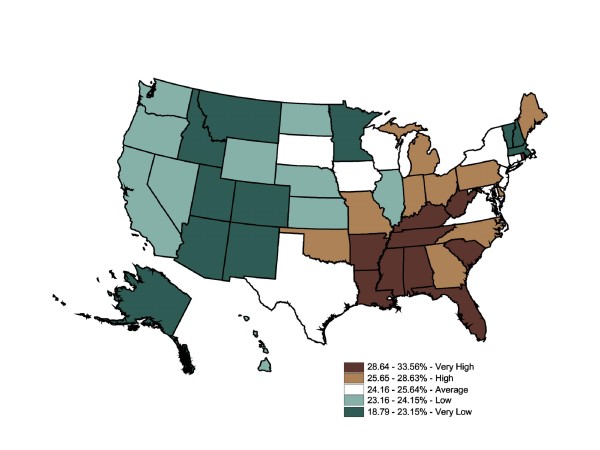
**State-Level Percentage of the Adult Population Told by a Doctor They Have Hypertension (BRFSS), 2003**. Note 1: Behavioral Risk Factor Surveillance System is used in the map. Note 2: Interpreted as the percent of the adult population told by a doctor they have hypertension in 2003. Note 3: 50 states and DC reporting. Note 4: Population 18+ is shown.

Figure 7 displays mapped BRFSS data. All 50 states and the District of Columbia administered this question in 2003. The map contains 10 states in each category except for the dark green (bottom 20%) category with 11. The state-level BRFSS self-reported prevalence ranged from 19% to 34%. This map shows 10 states ranked "very high" (29-34%) in self-reported hypertension prevalence rates. The remainder of the South and Central regions ranked as areas of "high" or "average" prevalence (24-29%). The majority of Midwest and Western states ranked as "very low" (19-23%) and "low" (23%-24%). The BRFSS mapped state-level data show hypertension prevalence rates were divided into high-prevalence Southeast and low-prevalence Midwest and West regions, with a cluster of very high-prevalence states roughly following the Appalachian region. The Global Moran's I for Figure 7 is 0.54, indicating low, positive spatial autocorrelation (p = 0.001).

**Figure 8:**
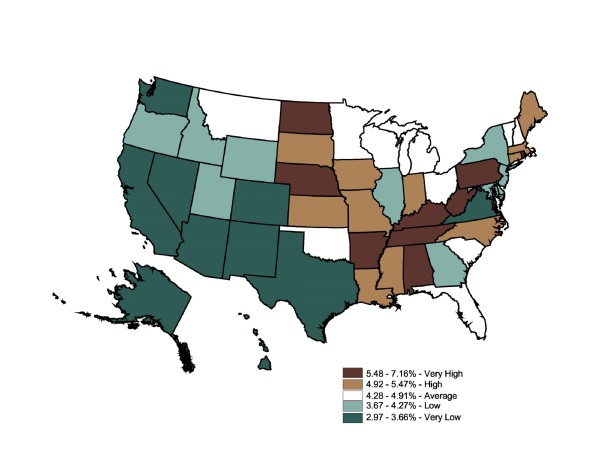
**State-Level Percentage of the Adult Population Who Filled Stroke Prescriptions, 2003**. Note 1: IMS Health Xponent is used in the map. Note 2: Interpreted as the percent of the adult population who filled stroke prescriptions in 2003. Note 3: 50 states and DC reporting. Note 4: Population 18+ is shown.

In comparison, the IMS Health state-level prescription map (Figure 8) showed stroke prescriptions filled shifting to the Midwest, while the Southeast had a less distinct pattern of very high and high rates of stroke prescriptions filled. Six states shifted down (from self-reported stroke to prescriptions filled). Residents of these states may be at risk for undertreatment by drugs for hypertension. This methodology cannot answer the public health question of health care access, but the inconsistency between the two maps is suggestive.

Shifts in the opposite direction were also seen in eight states. Perhaps more residents in these states filled hypertension prescriptions for treatment of their chronic disease compared to residents in other states. Likewise, the proportion with hypertension treated with prescriptions to those with diagnosed hypertension may be unusually high. We cannot ascertain the cause of this shift using current data. The Global Moran's I for Figure 8 is 0.37, indicating little spatial autocorrelation (p = 0.001).

**Figure 9:**
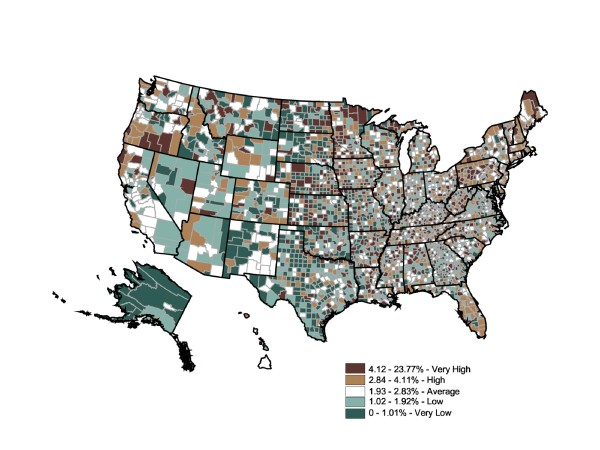
**County-Level Percentage of the Adult Population Who Filled Stroke Prescriptions, 2003**. Note 1: IMS Health Xponent is used in the map. Note 2: Interpreted as the percent of the adult population who filled stroke prescriptions in 2003. Note 3: 50 states and DC reporting. Note 4: Population 18+ is shown.

Figure 9 shows a varied county-level distribution of risk for stroke prevalence (expressed by prescription fill rates), ranging from 0% to 16% (two counties with rates above 15.8% were interpreted as regional distribution centers. The reported stroke prescription fill rate is 23.77% for Montour County, PA, and 19.01% for Adams County, ND. The remainder of the top quintile counties falls within the range of 4.12% and 15.75%).

Striking intercounty differences in prescription fill rates were found within the "very high" prevalence states, as well as in the "very low" prevalence states. The most informative patterns would be low prescription-fill-rate counties in states with overall high prevalence rates, high prescription-fill rate counties in states with overall low prevalence rates, and "very high" or "high" prescription-fill rate counties adjacent to "low" or "very low" prescription-fill rate counties. The Global Moran's I for Figure 9 is 0.03, indicating an absence of spatial autocorrelation (p = 0.001).

## Discussion

Based on the overall correlation results, we argue that prescription rates have the potential to be a useful and informative proxy for disease-specific diagnosed prevalence at both the state and county levels. Although we did not begin with *a priori *correlation goals, the results are sufficiently encouraging to warrant further investigation. With refinement of the methodology (see Future Directions below), we envision a powerful tool for health care planners, especially in rural areas. We did have an *a priori *expectation to see spatial autocorrelation (ie, geographic clustering) in rural areas because of the widespread belief that rural areas are at a disadvantage with respect to access to health care. However, this was clearly not the result, suggesting greater access to prescription drugs in rural areas than was originally thought. Finding no spatial autocorrelation at the national level is informative. But equally useful and not yet analyzed is spatial autocorrelation within states or regions that could prove enlightening to state health officials. Also unexamined are the geographic changes over time at the state, regional, and national levels. This methodology shows promise, especially for those individuals who are responsible for addressing the allocation of health resources and reducing health inequalities. This methodology's greatest potential is in providing a measure of diagnosed prevalence for rural counties, areas not sufficiently sampled in national surveys.

### Limitations

The progression from illness to prescription treatment is a series of steps - at any point, an individual can abandon the progression. Briefly, these steps include: (a) the patient's recognition of a medical need, (b) the patient decides to seek medical care, (c) the patient has access to medical care (overcoming physical, temporal, financial, and social constraints), (d) the patient is diagnosed, (e) the appropriate treatment includes a prescription drug, (f) the patient has access to prescription drugs (overcoming physical, temporal, financial, and social constraints), and (g) the patient fills the prescription and refills it regularly, having taken it as prescribed. However, to have been diagnosed, a patient must meet the first five of these steps. Thus, using prescription drug data to estimate prevalence is risky only in that the patient must have access to prescriptions and take them as prescribed, refilling regularly (not sharing or skipping doses).

The single biggest limitation to this methodology is that data regarding prescription drugs filled is limited to diagnosed and treated disease. Several studies have supplemented the diagnosed diabetes data in NHANES ("... have you even been told by a doctor or health professional that you have diabetes or sugar diabetes?") with blood samples drawn from the respondents (a fasting plasma glucose level of >126 mg/dL was coded as having diabetes). In a study of 1992-2002 NHANES data, Cowie et al found that some 30% of the nation's crude prevalence of total diabetes was undiagnosed [49], based on the additional blood tests. This finding was echoed in a study using 2003-2006 NHANES data by Danaei et al, [50] who found that 32% of total national diabetes is undiagnosed. A slightly higher percentage, about 40%, was found by Cowie et al using 2005-2006 NHANES data and blood tests [51]. As a methodological extension, Danaei et al applied their NHANES analysis methodology to state-level BRFSS data (2003-2007). They were able to report undiagnosed diabetes at the state level by age, sex, race, and insurance status. While this is an advance in terms of smaller geography (state vs. national), the BRFSS data are limited to state or large metropolitan areas. Conversely, since the prescription dataset does not contain demographic data, a further extension of Danaei et al's methodology is not feasible for county-level analysis. Researchers have also used the family history data in NHANES to estimate undiagnosed and pre-diabetes; however, this methodology is limited to national results [52-55].

The BRFSS survey data also have their own limitations in that individuals must have a landline telephone to participate; must be randomly chosen to participate; must answer the surveyor's call; must agree to participate if they do answer the phone; and must respond accurately to health status questions (ie, no faulty memory, avoidance of questions, etc.).

Measuring prescription-fill rates to use as a proxy measure of the prevalence of specific chronic illnesses is a crude methodology, fraught with possible disconnects, as the list above suggests. Nonetheless, we have accomplished an important validation exercise. Beyond what is listed above, we are aware of the coverage limitations of this prescription dataset, specifically the selection of the basket of drugs used to treat each disease. More specifically, we worked with IMS Health to create a basket of drugs that represents the best practices for the time period studied. IMS Health is the leading firm in the collection, analysis, and dissemination of prescription drug data in the US. They are aware of prescribing practices and trends in prescription drugs. Because the match between drug and illness is as much knowledge of what is occurring in the industry as it is the current medical guidelines, at the onset of the project, and very specifically, we chose IMS Health to select drug classes. Based on the objectives for the research and the specific disease states it planned to address, IMS recommended specific categories of drugs to include in the data extracted and provided. Though treatment practices change, most evidence indicates that they change slowly and should not have a dramatic effect on what we have presented here. Finally, when flows of individual drugs are examined, altering one or two in the calculations would have no effect on the overall conclusion of this manuscript, which is a new and potentially valuable, albeit imperfect, methodology to measure population health.

These issues need to be addressed in future refinement of this methodology. Indeed, many drugs are used to prevent or slow the onset of chronic illness. One future solution might be to link prescription-filled data with a survey of physicians' prescribing practices (e.g., of your patients for whom you prescribe heart disease medications, what percentage are preventative versus treatment for existing disease?).

The BRFSS and the IMS Health data measure slightly different things. Limitations of the BRFSS measure include recall bias, as well as lack of data on whether the respondent still had the disease or whether they were treating it with medications. The IMS data measure those who are treating their disease with medication due to disease progression, in conjunction with the ability to pay for care and treatment.

The correlations in 1999 were consistently lower than in later years. The base population in 1999 was a population estimate--as opposed to a census count, which occurred in 2000 --which may partially account for the differing magnitude between 1999 and subsequent years. The percentage change in population between 1999 and 2000 was as high as 12% in some counties, whereas in year-to-year comparisons (2000-2003), the state population differed by no more than 4%.

The national estimates from surveys were higher than prescriptions-filled rates due to incremental losses in the base population. There are several reasons prescriptions might not be counted. First, there are people outside the medical system, whether they excluded themselves prior to diagnosis or at the point of the doctor's office (those who were not diagnosed or not treated) or possibly at the point of the pharmacy (individuals who did not fill their prescriptions). A second category of exclusion was the distribution of drugs used in a clinical setting, such as a hospital (e.g., emergency room or surgery), clinic (e.g., chemotherapy and radiation) or the doctor's office (e.g., samples), although this would account for only a small percentage of chronic disease drugs, as opposed to other drug categories. The third category of exclusion from the prescriptions-filled dataset is nonparticipation in the prescription tracking program by the pharmacy or by the patient (e.g., mail-order purchases or purchases made outside the US); however, IMS Health accounts for this exclusion through estimation of the total prescriptions for the retail channel.

Additionally, all prescriptions were not filled in the county of patients' residence, although no test has yet been made of what percentage of prescriptions were filled outside of the county of residence [56]. Finally, there is the issue of off-label usage; we assume people who fill chronic disease prescriptions have that disease, as off-label usage is not perceived to be an issue with these particular classes of drugs.

Variations between self-reported prevalence rates of BRFSS and those currently in drug therapy for those diseases remain unaccounted for and are likely due to the BRFSS method of oversampling densely populated metropolitan areas (with the IMS data capturing all prescriptions) and variation between the rate of self-reported prevalence versus drug therapies. In other studies, the differences between treatment patterns [57], prescribing practices, and dispensing practices [58] have all been noted in state-level studies [59]. Finally, this methodology begs further exploration and refinement. We paired drug classes with appropriate BRFSS questions, but those choices can be challenged and should be tested.

## Conclusions

We tested the IMS database as a proxy for BRFSS prevalence, using diabetes, heart disease, and stroke as test chronic health conditions. Validated, this methodology can provide an efficient and timely county-level chronic disease surveillance system. Ultimately, the data can be used to detail the spatial characteristics of diabetes, heart disease, and stroke while investigating correlations among ecological, socioeconomic, and environmental conditions as a means of further exploring the social determinants of these chronic illnesses. Our spatial analyses indicate vast county-level variation in each of these prescription drug classes--variations that will need to be taken into account in future research as well as in the distribution of health-related resources.

### Future Directions

This area of inquiry affords many opportunities and issues for further exploration. To improve the accuracy of our estimates, (ie, county-level prevalence rates), the "cohort change ratio" technique could be employed rather than using US Census Bureau estimates [60,61]. Also, additional comparisons with other national health surveys, comparisons against regional- or county-level prevalence (if such data exist), and with a health provider's dataset (e.g., Blue Cross & Blue Shield or Express Scripts) should be explored. Finally, future researchers should seek a better calibration and correlation between drug classes and chronic illness, especially as they relate to heart disease and stroke. This could be informed via a survey of physician prescribing habits as well as disaggregating drug classes to test for correlations to specific illness, although this would not control for how treatment therapies change over time, nor the geographic lag that may exist for treatment regimen change.

## Competing interests

The authors declare that they have no competing interests.

## Authors' contributions

REC developed the concept, collected portions of the data, participated in the analysis, and initiated the initial and subsequent drafts. JSC conceived the concept, participated in the analysis, and substantially revised the manuscript drafts. WLJ refined the concept, participated in the analysis, led the spatial investigation, and revised manuscript drafts. TB interpreted the data and spatial analysis and provided critical commentary to subsequent drafts. RT devised measurement methodology and data sources and provided substantial methodological comments on the drafts. LGP refined measurement methodology and data sources and provided substantial methodological comments on the drafts. AGC refined the concept and measurement, participated in the analysis, and contributed substantial input on the implications. All authors reviewed and approved the final manuscript.
